# Cardiovascular and diabetes research in Africa to benefit from Servier sponsorship

**Published:** 2010-08

## Introduction

South African experts in the field of cardiovascular and diabetes medicine have recently been awarded significant support by Servier Laboratories, South Africa, to provide missing vital data on these conditions in African communities.

Servier has contributed to three initiatives, one being the establishment of a cardiovascular research chair in the Department of Medicine, Faculty of Health Sciences, University of Cape Town. The first incumbent of this clinical and research chair is Prof Karen Sliwa.

‘I hope during this tenure to focus on both laboratory-based and clinical cardiology research and provide novel insights into the mechanisms of cardiovascular conditions such as, for example, peripartum cardiomyopathy’, said Prof Karen Sliwa, newly appointed professor of cardiovascular research and director of the Hatter Cardiovascular Research Unit and also in a joint appointment at the Institute for Infectious Diseases and Molecular Medicine, University of Cape Town.

‘Servier’s support of this joint position is by means of an unconditional grant and this is a very welcome development, as longterm grants of this nature occur infrequently in African countries and in South Africa’, Prof Sliwa acknowledged.

Prof Sliwa is a specialist physician and cardiologist, holding a PhD from the University of the Witwatersrand. In addition, she is a specialist in tropical diseases, having completed a diploma in tropical medicine (DTM&H) and her doctoral thesis on Leishmanaisis at the Hadassah University in Jerusalem, Israel.

Drawn to the Hatter Institute because of its excellent laboratory facilities and research and educational activities established by Prof Lionel Opie over many years, Prof Sliwa has also initiated a new clinic with both the Department of Cardiology, under Prof Patrick Commerford’s leadership, and the Department of Obstetrics and Gynaecology, to provide a service for women with a cardiac condition who become pregnant and develop heart disease during/following pregnancy.

## Diabetes research benefits from Servier support

As part of this ongoing commitment to medical research in South Africa, Servier has funded a second prevalence and first trend survey of diabetes among urban Africans in South Africa. The last time a study of this kind was undertaken was in the 1990s.

A further Servier-sponsored study will be undertaken in KwaZulu-Natal to determine the genetic basis of diabetes among the Zulu community and to provide essential information on the genetic vulnerabilities and trends in diabetes among this South African community.

Steve Speller, CEO of Servier South Africa stressed the importance of this initiative. ‘From Servier’s perspective, the support of research into diabetes and cardiovascular disease, be it epidemiological, clinical or laboratory based, remains an important priority for the company. The increased incidence of diabetes in developing countries such as South Africa has reached almost epidemic proportions and research is therefore not only important to better understand how to treat diabetes, but also to understand the magnitude of the problem so that resources can be allocated appropriately.’

## Diabetes prevalence study in urban Africans

This prevalence study was initiated as a collaborative undertaking by UCT’s Department of Medicine and the MRC Research Unit for Chronic Diseases of Lifestyle. The study is being lead by Prof Naomi Levitt (UCT), with Dr Krisela Steyn and co-workers from both institutions.

Data collection for this study has already been completed from among the representative sample of 25- to 74-year-old urban Africans, randomly selected from the townships of Langa, Guguletu, Crossroads, Nyanga and Khayelitsha. More than a thousand participants were examined for the presence of diabetes and other cardiovascular risk factors.

Commenting on the relevance of this study, Prof Levitt pointed out that there are no available data that track the trends in diabetes prevalence in urban South Africans. ‘With increasing obesity and rapid urbanisation, with its accompanying lifestyle changes and increases in psychosocial stress, diabetes is highly likely to be on the increase in our African communities. This Servier-sponsored study will be indicative of prevalence changes among urban black South Africans in other major urban settings across the country and will provide vital data for policymakers in Government’, Prof Levitt stressed.

## Genetic determinants of type 2 diabetes in black South Africans

A large study of the genetic basis of type 2 diabetes among black South Africans has been initiated at the University of KwaZulu- Natal with financial support from Servier Laboratories, South Africa.

This is an important study as there is currently very little data available on the genetic polymorphisms that predispose or protect black Africans from developing type 2 diabetes. Equally, there is not a great deal known about genetic polymorphisms in type 1 diabetes in black South Africans, although Dr Fraser Pirie and co-workers have identified a polymorphism in the TLR 3 gene which may be associated with type 1 diabetes in South Africans of Zulu descent in KwaZulu-Natal.^1^

Smaller studies of black South Africans with type 2 diabetes have been undertaken by Dr Pirie and colleagues to identify whether genetic variants identified in European subjects could be detected in Indian and African subjects ‘These studies are small and statistically underpowered, but have indicated that these variants present in Caucasians are not well represented in our African population’, Dr Pirie pointed out.

The current study will be a full genome study of 1 500 type 2 diabetic patients and 1 500 controls from the African population of Zulu descent in KwaZulu-Natal. Collaboration with Oxford University will allow this large-scale genetic study to be done using automated genetic profiling. ‘With 3 000 samples being evaluated, we should be able to determine which polymorphisms occur commonly in our population and relate these variants/loci to the occurrence of type 2 diabetes, hypertension and obesity’, Dr Pirie said.

There are similar studies being undertaken in Uganda with support from universities in the UK and this will provide additional insights on African communities. ‘We are hoping to complete the study in the next two to three years and provide unique data of significant value to our South African and African communities’, Dr Pirie concluded.

This study will provide vital data, which is currently lacking, as to the genetic basis of diabetes pertinent to African populations.
